# Pasireotide treatment in Cushing’s disease: A single tertiary center’s experience

**DOI:** 10.3906/sag-2105-261

**Published:** 2021-12-18

**Authors:** Serdar ŞAHİN, Gular KARİMOVA, Şeyda Gül ÖZCAN, Emre DURCAN, Hande Mefkure ÖZKAYA, Pınar KADIOĞLU

**Affiliations:** Department of Internal Medicine, Faculty of Medicine, İstanbul University-Cerrahpaşa, İstanbul, Turkey

**Keywords:** Cushing’s disease, pasireotide treatment, hyperglycemia, hypercortisolism, UFC

## Abstract

**Background/aim:**

Cushing’s disease (CD), which cannot be controlled by surgery, requires medical treatment. In this situation, treatments with long-term effectiveness and safety profiles are needed. We aimed to evaluate the effects and adverse effects of pasireotide treatment in CD.

**Materials and methods:**

Patients who were followed up for CD and treated with pasireotide between 2014–2020 at Cerrahpaşa Medical Faculty, were evaluated retrospectively. The efficacy and adverse effects of pasireotide were evaluated in this study.

**Results:**

Thirty-two patients were evaluated. The mean duration of treatment was 26.5 [range, 12.0–37.0] months. The 24-h urinary free cortisol (UFC) decreased 46% during the treatment and normalized in 37.5% of patients. A significant decrement was found between pretreatment and last follow-up UFC (p = 0.001). Plasma ACTH decreased by 21%. A significant decrement was found between pre-treatment and the 3rd month, 6th month, and last follow-up ACTH levels (p = 0.014, p = 0.017, and p = 0.017, respectively). Serum cortisol levels decreased by 18% and a significant decrement was found between pretreatment and the 3rd month, and between pretreatment and the last follow-up (p = 0.034 and p = 0.013, respectively). While fasting blood glucose at the 3rd month was significantly higher than pretreatment fasting blood glucose, no significant difference was found between pretreatment fasting blood glucose and 6th month and last follow-up fasting blood glucose. Although there was a significant difference between pretreatment HbA1c levels and the HbA1c levels at the 3rd month (5.9% vs. 6.6% p = 0.007), 6th month (5.9% vs. 6.7% p = 0.003), and the last follow-up (5.9% vs. 7.1% p = 0.001), in the last follow-up, the majority (77%) of patients had adequate glycemic control (HbA1c ≤ 7.0 %).

**Conclusion:**

Pasireotide treatment is an alternative treatment in CD, remission is obtained in the first months of treatment, and continues for an extended period. Although hyperglycemia is the most common adverse effect, it can be successfully controlled.

## 1. Introduction

Cushing’s disease (CD) is a rare endocrine disorder associated with severe mortality and morbidity [1]. The annual incidence of CD is between 1.2 and 2.4 cases per million [2–4]. The most common clinical features of CD include obesity, diabetes, hypertension, moon facies, and facial plethora [5,6]. Diabetes, cardiovascular events, immunosuppression, osteoporosis, and psychiatric disturbances are associated with morbidity in patients with CD [7]. The estimated standard mortality rate for untreated patients with CD is 1.9%–4.8% [8–10]. Although mortality of patients with hypercortisolism in remission is lower than that of patients with persistent hypercortisolism, it is still high in these patients compared with the general population [11,12].

The primary treatment for CD is pituitary surgery. However, the remission rate ranges from 25% to 100% with expert pituitary surgeons, and the relapse rate is around 50% [13–17]. Resurgery, radiotherapy, bilateral adrenalectomy, and medical treatment are among the treatment options for patients with uncontrolled or relapsed CD [18]. Resurgery may not be appropriate in some patients because of high surgical risk, unresectable tumor, high hypopituitarism risk, and lower remission rates [19,20]. The other treatment option is radiotherapy, but its therapeutic effect is delayed and there are important central nervous system adverse effects [21]. Bilateral adrenalectomy requires lifetime use of glucocorticoid and mineralocorticoid therapy and carries the risk of Nelson syndrome [17].

Among the medical treatment options are ketoconazole, mitotane, metyrapone, mifepristone, dopamine agonists, and somatostatin receptor ligands (SRL) [1,18]. When evaluated in terms of their mechanism of action, there are dopamine agonists and SRLs among drugs that act on corticotroph cells. SRLs with high affinity for somatostatin receptors (SSTRs)-2, such as octreotide and lanreotide, are used effectively in the treatment of acromegaly and gastroenteropancreatic neuroendocrine tumors, but they have been unsuccessful in their use in CD due to their medium and low affinity for the remaining SSTRs subtypes [22, 23]. In vitro studies, strong SSTR-5 expression was shown in corticotropic tumor cells in CD. SSTR-5 expression is unaffected by hypercortisolemia, while SSTR-2 expression is suppressed but can upregulate with eucortisolemia [24,25]. Pasireotide binds with high affinity to SSTR-1, SSTR-2, SSTR-3, SSTR-5, and also binds to SSTR-5 with 40-fold higher affinity than octreotide [24,25]. SSTR-5 expression in corticotropic tumor cells and high affinity of pasireotide may explain the potential effect of pasireotide on CD. In this study, we aimed to evaluate the long-term effects and adverse effects of pasireotide treatment.

## 2. Materials and methods

### 2.1. Participants

Patients who were followed up in Cerrahpaşa Medical Faculty and received pasireotide treatment between 2014–2020 were evaluated. The patients’ data included in the study were retrospectively collected from their medical records. Patients who were aged over 18 years and received pasireotide treatment were included. Patients who had a risk of long QT syndrome, uncontrolled diabetes, liver and kidney failure, and pregnant patients were excluded. The patients were retrospectively evaluated pretreatment, at the 1st, 3rd, and 6th months, and the 1st year of the treatment, and 1-year intervals thereafter.

### 2.2. Clinical evaluation

Clinical parameter evaluation of each visit was retrospectively recorded. The clinical parameters included systemic arterial hypertension, weight, myopathy, and plethora. Normalization of arterial hypertension, weight reduction, and regression of myopathy was accepted as clinical improvement. According to the guidelines of the American Society of Hypertension and the International Society of Hypertension, systolic blood pressure ≥ 140 mmHg and/or diastolic blood pressure ≥ 90 mmHg or treatment of a previously diagnosed hypertension was defined as systemic arterial hypertension [26]. The patients’ age, sex, total disease duration, preoperative adenoma size, Crooke cell presence, Ki-67 labeling index, the number of surgical procedures, and the presence of radiotherapy were evaluated. The tumor size was evaluated using the largest tumor diameter on magnetic resonance imaging (MRI).

### 2.3. Evaluation of pasireotide treatment

The effectiveness of pasireotide treatment was evaluated using basal serum cortisol, plasma adrenocorticotropic hormone (ACTH) level, salivary cortisol, 24-h urinary free cortisol (UFC), the upper limit of normal (ULN) for UFC, duration and doses of pasireotide treatment, the time between pituitary surgery, pituitary hormone status after pituitary surgery, and initiation of pasireotide and clinical parameters. Patients with UFC ≤ ULN and clinical improvement were defined as controlled, patients with a UFC ≥ ULN but with a decrease of more than 50% and clinical improvement were defined as partially controlled, and the other patients as uncontrolled. Patients who received radiotherapy before or during pasireotide treatment or who underwent surgery during pasireotide treatment were considered uncontrolled patients.

### 2.4. Safety analysis

Patients were evaluated based on their medical records in terms of adverse effects that occurred due to pasireotide treatment. Antidiabetic agents used in the treatment of type 2 diabetes (T2DM), fasting blood glucose (FBG), and glycated hemoglobin (HbA1c) values, hepatobiliary ultrasonography findings, liver enzymes [alanine aminotransferase (ALT), aspartate aminotransferase (AST)], and electrocardiography (ECG) findings were evaluated.

### 2.5. Assays

FBG and liver enzymes (ALT, AST) were analyzed using Roche/Hitachi Cobas c systems according to the manufacturer’s kits and recommendations (Roche Diagnostics GmbH, Mannheim). The HbA1c levels were measured using an International Federation of Clinical Chemistry (IFCC)-certified automated high-performance liquid chromatographic method (Lifotronic H9, M/s SVR Biotech, Hyderabad, India). Salivary cortisol was measured using an automated electrochemiluminescence immunoassay (ECLIA) (Roche; Cobas e 601). Plasma ACTH, serum cortisol, and UFC were assayed using an ECLIA method with Roche Cobas e systems (Roche, Cobas e 602, Roche Diagnostics GmbH, Mannheim, Germany).

### 2.6. Statistical analysis

The data were analyzed using the Statistical Package for the Social Science program, SPSS 25.0 (SPSS, Inc, Chicago, IL, USA). The findings of the study are presented as numbers and percentages for categorical variables, and mean, standard deviation, median, minimum, and maximum for continuous variables. Comparisons between categorical variables such as disease control rate (among groups with mild, moderate, and severe disease severity), sex, and disease severity (between control and uncontrolled groups) were made using the chi-square (χ2) test. The distribution of variables was examined using the Shapiro-Wilk test. We compared the parameters with normally distribution pre and posttreatment with the paired-samples t-test, and the parameters with nonnormally distribution with the Wilcoxon signed-ranks test. In addition, between controlled and uncontrolled groups, the parameters with normally distribution were evaluated with an independent sample t-test, and the parameters with nonnormally distribution with the Mann-Whitney U test. In correlation analysis, Pearson’s correlation coefficient was used in the case of normally distributed data, and Spearman’s correlation coefficient was used with nonnormally distributed data. Statistical significance was set as p < 0.05 with a confidence level of 95%.

This study was approved by the local ethics committee (approval number: 77992). The study adhered to the tenets of the Declaration of Helsinki.

## 3. Results

### 3.1. Demographic data

The medical records of 201 patients with CD followed in Cerrahpaşa Medical Faculty were retrospectively reviewed. Thirty-four of 201 patients with CD were receiving pasireotide treatment. Thirty-two of 34 patients treated with pasireotide were evaluated. Two patients were excluded because they did not attend regular follow-up. Twenty-five (78%) of the patients were female, and seven (22%) were male. The mean age at the time of diagnosis was found as 39.75 ± 11.1 years. The median follow-up period was 86.0 [range, 55.0–130.0] months. The general features of the patients are shown in Table 1. The mean duration of pasireotide treatment was 26.5 [range, 12.0–37.0] months. The mean pasireotide initial dosage was 0.919 ± 0.37 mg/day, the initial dose of pasireotide was 0.6 mg/day in 17 patients, 1.2 mg/day in 13 patients, and 1.8 mg/day in two patients. The mean pasireotide dose at the last follow-up was 1.088 ± 0.50 mg/day. The last dose of pasireotide was 0.3 mg/day in two patients, 0.6 mg/day in 11 patients, 1.2 mg/day in 11 patients, and 1.8 mg/day in 8 patients. Postoperative data of 27 (84%) patients are available. FSH and LH deficiency was detected in two (7%) of these patients, and only LH (3%) deficiency was detected in one patient. Postoperative diabetes insipidus developed in one (3%) patient.

### 3.2. Effectiveness of pasireotide treatment

**Figure 1:**
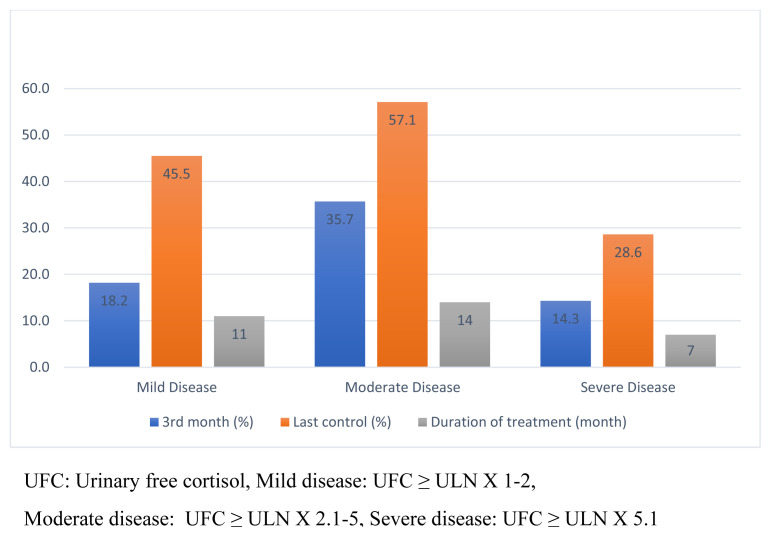
The percentage of patients whose disease was controlled at the 3rd month and last control, and duration of pasireotide treatment.

A decrease of 46% was found in the UFC values of the patients after pasireotide treatment. There was no significant difference between pretreatment UFC values and those at the 1st (151.0 [105.8–251.2] vs. 120.7 [57.4–264.4], p = 0.446), 3rd (151.0 [105.8–251.2] vs. 93.0 [51.3–220.5], p =0.114), and 6th (151.0 [105.8–251.2] vs. 113.0 [42.0–160.8], p =0.078) months. However, the last follow-up UFC was significantly lower compared with the pretreatment UFC (151.0 [105.8–251.2] vs. 68.7 [24.6–142.5], p = 0.001). UFC values were normalized in 12 (37.5%) patients (controlled disease), and a decrease of more than 70% was detected in the UFC of three (9%) patients (partially controlled disease). The remission rates at the 3rd month and last follow-ups are given in Figure 1. Although there was no statistical significance, the rate of disease control in severe disease was lower than in mild and moderate disease UFC (UFC ≤ 5 × ULN) both at the 3rd month and at the last follow-up.

In the patients whose UFC normalized, ketoconazole treatment was added instead of an increase in pasireotide dose due to uncontrolled hyperglycemia in the treatment of two patients. However, because of insufficient response, ketoconazole treatment was discontinued, and the dose of pasireotide treatment was increased, and hyperglycemia was controlled with insulin and oral antidiabetic agents. In 62.5% of the patients who were controlled in the 3rd month, the control status continued at the last follow-ups. In contrast, 41.6% of the patients who were not controlled at 3 months were controlled at the last follow-ups. According to the fit hypothesis analysis (Kappa test), the treatment response at the third month could not predict the response at the last follow-up (p = 0.274). After pasireotide treatment, a 21% decrease in ACTH, 18% decrease in serum cortisol, and 48% decrease in salivary cortisol values were found. In addition, there was a significant decrease in UFC, ACTH, and cortisol levels between pretreatment and the last follow-up, but no significant difference was found in salivary cortisol values. These parameters are shown in Table 2. An improvement in myopathy in 14 (43%) patients, an improvement in facial plethora in 3 (9%) patients, and a decrease in weight in 16 (50%) patients were detected. A significant decrease was found in patient weights between pretreatment and the last follow-up (81.7 ± 17.7 vs. 75.7 ± 15.1 kg, p = 0.016), and a mean difference in weights of 6 kg.

Twenty-four (75%) patients had a diagnosis of systemic arterial hypertension prepasireotide treatment, and eight (25%) patients did not. Twenty-one (87.5%) of the 24 patients diagnosed as having systemic arterial hypertension received medical treatment, three (12.5%) did not receive medical treatment prepasireotide treatment. At the last follow-ups, 18 (56%) patients were continuing medical treatment due to hypertension, and 14 (44%) patients had normal blood pressure without medication. In addition, medical treatments due to hypertension were discontinued in three (14%) of 21 patients after pasireotide treatment. There was a significant difference between the pretreatment and last follow-up systole and diastolic blood pressures of the patients (p = 0.016 and p = 0.026, respectively). The median systole/diastole blood pressures were 127.5 [118.7–140.0]/80 [70.0–80.0] mmHg pretreatment, and 120.0 [117.5–122.5]/70.0 [70.0–76.2] mmHg at the last follow-up. Systolic and diastolic blood pressure was <140/90mm Hg in all patients receiving medical therapy at the last follow-ups.

### 3.3. Predictive factors of pasireotide treatment response

The rate of controlled patients (total and partial controlled) was 15/32 (46%). No significant difference was found between controlled and uncontrolled patients in terms of age, sex, disease severity, initial plasma ACTH, initial serum cortisol, initial salivary cortisol, initial UFC levels, FBG (initial and 3rd month), and HbA1c values (initial and 3rd month). Furthermore, there was no significant difference in terms of pathologic features (Ki-67 labeling index, mitosis, presence of Crooke cells), adenoma size, weight change, total pasireotide treatment time, initial and last pasireotide treatment dose, the time between pituitary surgery and initiation of pasireotide treatment, and median systolic/diastolic blood pressures between the controlled and uncontrolled patients. The general features of controlled and uncontrolled patients are shown in Table 3

### 3.4. Adverse effects of pasireotide treatment

During the pasireotide treatment, gastrointestinal symptoms developed in three (9%) patients, and the drug was discontinued in one (3%) of these patients due to severe adverse effects. There was no significant change in the liver enzymes of the patients. Three (9%) patients underwent cholecystectomy during pasireotide treatment, two of whom had a history of cholelithiasis before the pasireotide treatment. During the pasireotide treatment, no serious QT prolongation was detected, but one patient with known coronary artery disease had acute myocardial infarction while taking pasireotide, and one patient was diagnosed as having angiosarcoma.

### 3.5. Glucose metabolism

**Figure 2:**
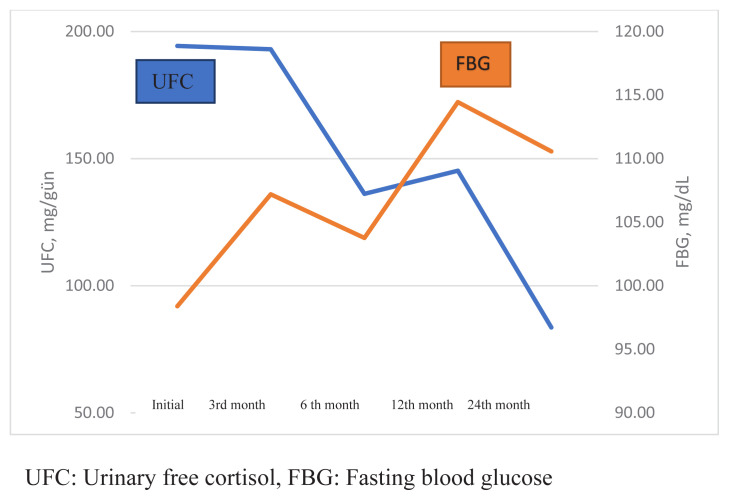
The association of UFC and FBG during pasireotide treatment.

**Figure 3:**
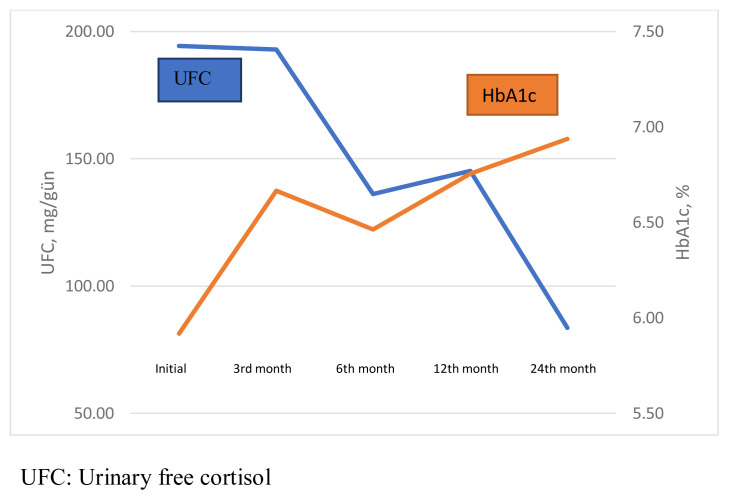
The associaton of UFC and HbA1c during pasireotide treatment.

At the last follow-up, a mean increase of 11.3 mg/dL in FBG and 1% in HbA1c levels was observed. Although there was a significant increment between the pretreatment and 3rd-month FBG (p = 0.008), no significant difference was found between pretreatment and 6th month and last follow-up FBG (p = 0.126 and p = 0.070, respectively). There was a significant increment between pretreatment HbA1c values and the 3rd-month (5.8 [5.4–6.3] vs. 6.4 [5.9–7.0], p = 0.007), 6th-month (5.8 [5.4–6.3] vs. 6.6 [6.2–7.5] p = 0.003), and last follow-up HbA1c values (5.8 [5.4–6.3] vs. 5.9 [6.3–8.4], p = 0.001). No significant correlation was found between the 3rd-month UFC (93. [51.3–220.5]) and 3rd-month FBG (101.0 [88.5–122.5]), (r = 0.023, p = 0.935), but there was a positive correlation between the 3rd-month UFC (93. [51.3–220.5]) and 3rd-month HbA1c (6.4 [6.0–7.1]) (r = 0.513, p = 0.029).The associations between UFC–FBG and UFC–HbA1c during pasireotide treatment are shown in Figure 2 and 3. Prepasireotide treatment, 11 (34.3%) patients had a diagnosis of T2DM, and seven (21.8%) patients had a prediabetes diagnosis. Prepasireotide treatment, 10 (31.2 %) patients were using metformin, two (6.2 %) used DPP-4 (dipeptidyl peptidase-4) inhibitors, three (9.3 %) used basal insulin, one (3.1 %) used sulfonylurea. In the third month of treatment, T2DM developed in four (12.5%) patients with prediabetes, and prediabetes developed in four (12.5%) patients. In 21 of 27 (77%) patients who received treatment for T2DM, the target HbA1c values (≤7%) were reached at the last follow-up. At the last follow-up, 23 (71%) patients were using metformin, 18 (56%) used DPP-4 (dipeptidyl peptidase-4) inhibitors, two (6.2%)used glucagon-like peptide-1 (GLP-1) agonists, three (9%) used basal insulin, four (12.5%) used intensive insulin, one (3%) used sulfonylurea, and one (3%) patient was using a sodium-glucose cotransporter 2 (SGLT2) inhibitor.

## 4. Discussion

We observed significant improvements in the hypercortisolemia levels of the patients. No serious adverse effects were observed, except for glucose dysregulation. Although dysregulation in glucose was observed at the 3rd month of pasireotide treatment, the target HbA1c was achieved in most patients. There was a significant decrease in the weight of the patients.

The normalization rates of UFC with pasireotide treatment in CD range from 34.5% to 68.8% [27,28,29,30,31]. Previous studies confirmed that the rate of UFC normalization rate increased as the duration of pasireotide treatment was prolonged [29,30]. Another parameter affecting the UFC normalization rate is disease severity. Normalization rates were higher in patients with mild and moderate UFC values before pasireotide [27,30]. From this point of view, in our study, the UFC normalization rate was lower compared with previous studies. However, some points should be taken into consideration while evaluating these results. The rate of severe disease was high, and we did not include patients who received radiotherapy in this group, even if their UFC values returned to normal, regardless of the time of radiotherapy. In addition, in line with previous studies, our patients with mild to moderate hypercortisolemia responded better and faster to treatment. Pretreatment UFC values can guide in terms of patient selection. It may be more rational to consider pasireotide treatment mostly in patients with mild and moderate UFC values.

The prevalence of hypertension in CD is 55%–85% [32] and contributes to cardiovascular risk [33]. In a study by Pivonello et al., similar decreases in systolic and diastolic blood pressures were found postpasireotide treatment in controlled and uncontrolled patients [34]. Accordingly, the authors reported that pasireotide treatment improved hypertension independent of a cortisol reduction [34]. In this respect, similar results can be seen when looking at our results. Significant decreases were detected postpasireotide treatment in our patients, and there was no significant difference between the controlled and uncontrolled patients in terms of systolic and diastolic blood pressures. In addition, the blood pressures of all patients (controlled and uncontrolled patients) were found to be within the normal range at the last follow-ups. Thus, pasireotide treatment may improve hypertension independent of decreases in cortisol. However, studies involving larger numbers of patients are needed to clarify this issue.

Hyperglycemia is one of the important points to be considered, especially in the management of pasireotide treatment. It is seen that patients receiving pasireotide treatment need more antidiabetic drugs during the treatment process [27,30]. We also found similar results. However, in the majority of patients receiving pasireotide treatment, glucose dysregulation was under control in the early period. Similar to previous studies, hyperglycemia was controlled in the early phase of pasireotide treatment in our study [27–29,35,36]. In other words, although hyperglycemia seems to be an important problem in pasireotide treatment, it can be overcome with successful antidiabetic treatment.

Previous studies have attempted to clarify the mechanism of pasireotide-induced hyperglycemia. In a study in healthy volunteers, it was shown that pasireotide caused a decrease in incretin and insulin secretion, and did not cause any changes in insulin sensitivity [37]. Clinical studies also support this situation. Breitschaft et al. showed that vildagliptin and liraglutide were more effective in controlling pasireotide-induced hyperglycemia [38]. In our study, 56% of the patients who received antidiabetic treatment were using DPP4-inhibitors, and 6.2% used GLP1 agonists. However, the duration of the study by Breitschaft et al. was one week, and therefore, it was a short time for observing the long-term effects of antidiabetic agents in lowering pasireotide-induced hyperglycemia. Longer and prospective studies may shed light on this issue.

Our analysis showed that there was a positive correlation between the 3rd-month UFC and 3rd-month HbA1c. Although this result was not very strong, it supports that hyperglycemia was not solely due to the effect of pasireotide treatment, but also endogenous hypercortisolism because this affects postprandial glucose rather than FBG [39]. However, glycemic control was found independent of UFC responses in previous studies [27,30]. The hyperglycemia mechanism of pasireotide treatment was only performed on healthy volunteers [37]; therefore, the development mechanisms in the patient population are not fully known. For this reason, more studies are needed on this issue.

Gastrointestinal disorders associated with the pasireotide treatment are generally tolerable and temporary. In the first 12 months of the phase III study conducted by Colao et al., nine (6.6%) patients had bile sludge, 27 (19.7%) patients had gallstones, and six (3.7%) patients underwent cholecystectomy. Transient slight increases in liver enzymes were found in 29% of patients [27]. In another study, it was observed that the symptoms of patients with nausea (21.4%) and vomiting (14.3%) regressed spontaneously within a few weeks or months, only one patient was unresponsive to medical treatment, and two (14.3%) patients had diarrhea that improved with loperamide [36]. Similar results to previous studies were found in our study. Accordingly, although gastrointestinal symptoms do not cause serious problems during pasireotide treatment, they have an important place in treatment follow-up. In very few cases, discontinuation of pasireotide treatment or additional intervention may be required.

Our study has a few limitations. First, our sample size is small due to the rarity of the disease and the scarcity of cases suitable for treatment. Secondly, the data of some patients are lacking due to the retrospective design. Thirdly, the effect of pasireotide on adenoma could not be evaluated due to the lack of data.

In conclusion, pasireotide treatment is an alternative to other treatments in patients with CD. Patients who have controlled disease in the first months of treatment continue this situation for an extended period. Although glucose metabolism disorder is the most common adverse effect, it can be successfully controlled. Further and extensive studies are required to investigate factors that predict the success or failure of the treatment and to better understand the adverse effect mechanisms.

## Figures and Tables

**Table 1:** General features of patients.

The laboratory parameters prepasireotide treatment	

Serum cortisol (μg/dL) (n = 32) (mean ± sd)	18.1 ± 5.7

Plasma ACTH, (pg/mL) (n = 32) (mean ± sd)	48.1 ± 28.0

UFC, (mg/day) (n = 32) (mean ± sd)	189.7 ± 116.0

Salivary cortisol (μg/dL) (n = 11) (median [IQR])	0.34 [0.06–0.6]

The adenoma size in the initial pituitary MRI (n = 23) (mm) (mean ± sd)	7.1 ± 5.0

The adenoma in the initial pituitary MRI (n = 27)	

Microadenoma (n (%))	17(53.0)

Macroadenoma (n (%))	6 (18.8)

Absence of adenoma (n (%))	4 (12.5)

The pathology features	

The presence of Crooke cell (n (%))	11 (78.5)

Ki-67 % labelling index (n = 18) (median [IQR])	1.0 [1.0–3.0]

Surgery (number of operation)	
1 operation (n (%))	18 (56)
2 operation (n (%))	12 (37)
3 operation (n (%))	2 (6)

Radiotherapy (n (%))	8(25)

ULN (≥ULN) (n = 32) (disease activity according to UFC)	
Mild (≥ULN × 1–2) (n (%))	11 (34)
Moderate(≥ULN × 2.1–5) (n (%))	14 (43)
Severe (≥ULN × 5.1) (n (%))	7 (21)

**ACTH:** Adrenocorticotropic hormone, **IQR:** Interquartile range, **UFC:** Urinary free cortisol, **ULN:** Upper limit of normal

**Table 2:** The comparison of pretreatment and 3rd month, 6th month, and last control of the laboratory parameters.

	Pretreatment	3rd month	6th month	Last control
UFC levels (mg/day), median [IQR]	151.0 [105.8–251.2]	93.0 [51.3–220.5]	113.0 [42.0–160.8]	68.7 [24.6–142.5]
p		0.114	0.078	**0.001**
ACTH levels (pg/mL), mean ± sd	48.1 ± 28.0	38.3 ± 19.7	35.0 ± 18.8	38.0 ± 23.0
p		**0.014**	**0.017**	**0.017**
Baseline cortisol levels (μg/dL), mean ± sd	18.1 ± 5.7	15.2 ± 6.0	15.7 ± 5.7	14.8 ± 5.4
p		**0.034**	0.076	**0.013**
Salivary cortisol levels (μg/dL), median [IQR]	0.34 [0.06–0.6]	0.2 [0.1–0.4]	0.2 [0.07–0.4]	0.1 [0.1–0.3]
p		0.285	0.446	0.207

**ACTH:** Adrenocorticotropic hormone, **UFC:** Urinary free cortisol, **IQR:** Interquartile range.

**Table 3:** The comparison of demographic, clinical, and laboratory parameters of controlled and uncontrolled patients.

	Controlled patients (n: 15)	Uncontrolled patients (n: 17)	p
Sex (F (%)/M (%))	10 (66.7) / 5 (33.3)	15 (88.2)/2 (11.8)	0.209
Age (years)	39.5 ±10.5	39.9 ± 11.9	0.920
Pretreatment baseline cortisol (μg/dL)	17.8 ± 5.3	18.4 ± 6.2	0.680
Pretreatment ACTH (pg/mL)	40.0 [21.1–59.0]	54.0 [25.2–71.7]	0.350
Pretreatment UFC mg/day	147.0 [107.0–230.0]	155.0 [105.2–287.8]	0.911
Pretreatment salivary cortisol μg/dL	0.29 [0.06–0.58]	0.43 [0.12–3.4]	0.556
Pretreatment FBG mg/dL	90.0 [82.0–96.5]	82.5 [78.0–119.0]	0.746
Pretreatment HbA1c (%)	6.0 [5.5–6.3]	5.8 [5.4–6.3]	0.605
The adenoma size in the initial pituitary MRI (mm)	5.0 [4.0–9.2]	5.0 [3.7–11.0]	0.879
The presence of Crooke cell (n, %))	5 (71.4)	6 (85.7)	1.000
Ki-67 % labelling index (median [IQR])	1.0 [0.7–5.7]	2.0 [1.0–3.0]	0.660
Total pasireotide treatment time (months)	36.0 [12.0–44.0]	24.0 [9.0–36.5]	0.105
Pasireotide initial dose mg/day	1.2 [0.6–1.2]	0.6 [0.6–1.2]	0.576
Weight pretreatment	82.5 [65.0–109.5]	79.2 [70.0–87.0]	0.667

**F:** Female, **M:** Male, **ACTH:** Adrenocorticotropic hormone, **UFC:** Urinary free cortisol, **FBG:** Fasting Blood Glucose

**MRI:** Magnetic resonance imaging, **IQR**: Interquartile range
